# A national systematic literature review for aflatoxin M1 in commonly consumed cheese brands in Iran: Human health risk assessment by Monte Carlo simulation

**DOI:** 10.1016/j.heliyon.2023.e19679

**Published:** 2023-08-31

**Authors:** Tooraj Massahi, Amir Kiani, Kiomars Sharafi, Abdullah Khalid Omer, Gholamreza Ebrahimzadeh, Jalil Jaafari, Nazir Fattahi, Kimya Parnoon

**Affiliations:** a Student Research Committee, Kermanshah University of Medical Sciences, Kermanshah, Iran; b Regenerative Medicine Research Center (RMRC), Kermanshah University of Medical Sciences, Kermanshah, Iran; c Pharmaceutical Sciences Research Center, Health Institute, Kermanshah University of Medical Sciences, Kermanshah, Iran; d Research Center for Environmental Determinants of Health (RCEDH), Research Institute for Health, Kermanshah University of Medical Sciences, Kermanshah, Iran; e Department of Environmental Health Engineering, School of Public Health, Kermanshah University of Medical Sciences, Kermanshah, Iran; f Department of Food Hygiene and Quality Control, Faculty of Veterinary Medicine, Urmia University, Urmia, Iran; g Department of Environmental Health Engineering, School of Public Health, Zabol University of Medical Sciences, Zabol, Iran; h Research Center of Health and Environment, Guilan University of Medical Sciences, Rasht, Iran

**Keywords:** Aflatoxin M1, Cheese, Health risk assessment, Systematic review, Iran

## Abstract

Cheese is popular in Iran because of its high nutritional value; therefore, it is necessary to control this product regarding health risk factors, particularly aflatoxin M1 (AFM1). This research reviewed AFM1 in various varieties of cheese in Iran to assess the potential health risks associated with consuming these products for different age groups. In this regard, all accessible papers from different databases were screened between June 27, 2000 and October 10, 2022 b y systematic research and then considering the selection criteria of the studies; finally, 22 articles were selected for the current review. The amount and prevalence of AFM1 were calculated and separated based on the cheese variety, and the sampling location; health risk assessment (HRA), statistical, uncertainty, and sensitivity analysis for AFM1 of cheese for different age groups were performed. The study results for 2143 samples showed that the overall average AFM1 for cheese is 160 ± 175 ng/kg, below the European Commission (EC) regulation (250 ng/kg). AFM1 contaminated 72.42% of all cheese samples, and 13% of these contaminated samples had a higher AFM1 than the EC regulation. Cheese varieties were ranked based on average levels of AFM1 as white pasteurized > traditional > creamy > probiotic > Lighvan, and this ranking was obtained based on sampling locations as market > dairy factories > livestock farms. Based on the HRAs, from the perspective of the liver cancer risk (LCR), the margin of exposure (MOE), and the hazard index (HI) approach, it can be concluded that cheese produced in Iran, in terms of AFM1, particularly for children, poses serious health risks. Accordingly, it is imperative to carefully consider implementing suitable management methods to inhibit the growth of aflatoxin B1 (AFB1) in livestock fodder, and training in sanitary production and processing of dairy products according to world standards is suggested for industrial and traditional cheese producers across Iran.

## 1 Introduction

Mycotoxins are secondary toxic metabolites produced by fungi under appropriate temperature and moisture conditions in various agricultural and food products [1]. Of the 300 different mycotoxins, aflatoxins are the most poisonous and famous mycotoxins that have been extensively studied worldwide and are generally formed by certain species of *Aspergillus*, including *Aspergillus Parasiticus, A. Nomius, and A. flavus* [2,3]. These compounds are carcinogenic, mutagenic, and teratogenic and cause acute and chronic poisoning in humans [3,4]. Common forms of aflatoxin encompass B1, B2, G1, G2, M1, and M2 [2]; among them, AFB1 is the most commonly formed mycotoxin and has been identified as the most potent naturally occurring carcinogen in mammals [5,6].

AFM1 is a monohydroxylated derivate of AFB1 [7]. When animals ingest AFB1-contaminated feed, the toxin is metabolized to AFM1 by the cytochrome P450 enzyme system in the animal's liver and then excreted in milk [8]. Depending on various factors, including order and stage of lactation, individual response, and ingestion rate of AFB1, around 0.3–6.2% of AFB1 in livestock forage is converted to AFM1 in milk [9,10]. Although AFM1 has less toxicity than AFB1, its genotoxic, cytotoxic, and carcinogenic effects have been well documented. Therefore, the “International Agency for Research on Cancer (IARC) has classified AFB1 and AFM1 as group 1 human carcinogens” [11,12].

The effects of aflatoxins on humans are indirectly experienced by consuming animal products, especially dairy products such as cheese [8,13]. Research has indicated that the existence of AFM1 in milk and dairy products presents a considerable risk to public health, particularly in communities where dairy consumption is a prominent component of their daily nutritional intake [14,15]. Sterilization, pasteurization, preparation, and storage techniques in the dairy industry and milk processing for cheese production fail to impact the reduction of toxicity and survival of AFM1 significantly. Furthermore, the concentration of AFM1 remains unchanged during these processes [16,17]. It was also recently confirmed that AFM1 maintains stability throughout many cheese varieties' ripening and storage steps [2].

The incidence of aflatoxin in cheese can be attributed to several possible factors, including 1) it can occur when animals are fed with feed containing AFB1, leading to the contamination of milk used in cheese production with AFM1. This is a significant contributor to the presence of AFM1 in cheese [13], 2) growth of *A. parasiticus* and *A. flavus* on cheese can result in the synthesis of aflatoxin (B1, B2, G1, and G2) [2], and 3) using powdered milk contaminated with aflatoxin during the cheese-making process to enrich milk can also contribute to the presence of AFM1 in cheese [2].

Limits on AFM1 in milk and its products have been recommended in several countries to keep customers, especially kids. These guidelines vary according to economic and other factors in various nations, affecting the average contamination intake [9,15,16]. Therefore, the European Commission (EC) and the Iranian Standard and Industrial Research Institute (ISIRI) have set the maximum limit of AFM1 (250 ng/kg) in different types of cheese [18,19].

Due to the significance of milk and its products in human nutrition and health, their widespread availability and the possibility that they contain aflatoxin residues are a global concern. Therefore, their health and safety should be prioritized [2,20]. The occurrence of AFM1 in dairy products poses a significant health concern in developing nations, including Iran [20]. Therefore, monitoring and providing control strategies to reduce this contaminant in dairy products is crucial.

The investigation of AFM1 occurrence in dairy products in various regions of the world has been the subject of numerous food safety studies. In this regard, various studies in Iran have revealed the presence of AFM1 in various cheeses. Some results are consistent with each other, and others are not. Therefore, it is necessary to summarize the results of the studies mentioned above to determine the health status of different types of cheeses ingested in Iran in terms of AFM1.

In Iran, in recent years, minimal reviews have been performed on evaluating AFM1 in some dairy products, such as milk [15,[21], [22], [23], [24]]. There is no systematic review to summarize findings and offer a comprehensive assessment of the level of AFM1 contamination and the health risk assessment of other popular dairy products, including cheese. The present study used a systematic review to estimate the level of AFM1 in different kinds of cheese in Iran and evaluate the HRA of this product. To comprehensively estimate the health risk associated with consuming AFM1-contaminated cheese, we used Monte Carlo simulation, a widely statistical method for estimating the distribution of exposure to mycotoxins in food and quantifying related health risks. The results of this study can offer valuable insights into the current levels of contamination of different types of cheese with AFM1 and the potential risks posed to public health by consuming AFM1-contaminated cheese. These findings can facilitate the development of policies and decisions related to food safety and public health protection on a global scale.

## 2 Materials and methods

### 2.1 Search strategy

**Fig. 1:**
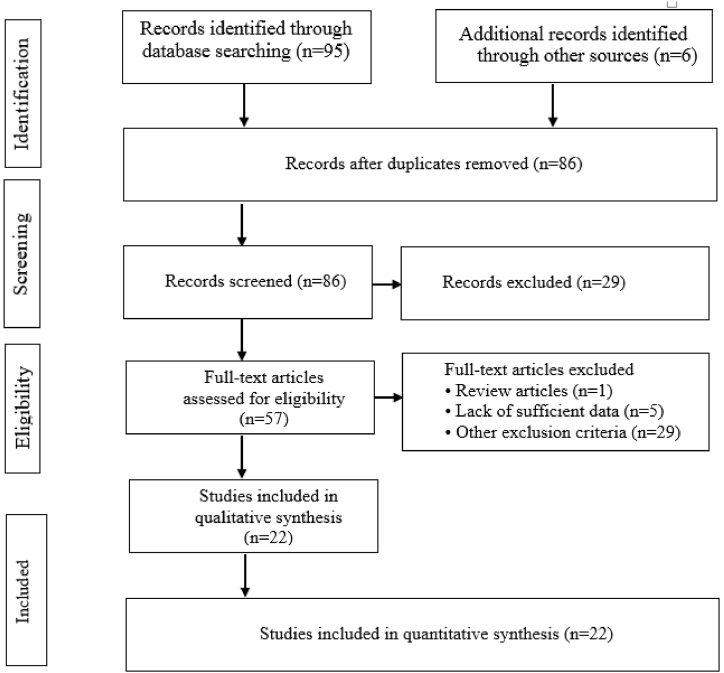
Flow diagram of study identification according to PRISMA.

A systematic search of available international databases (Scopus, Science Direct, Web of Science, PubMed, and Google Scholar) and national (Iran doc, SID, Magiran, etc.) was conducted between June 27, 2000 and October 10, 2022. Reference checking and citation tracing were conducted as part of the study to reduce the probability of unintentionally eliminating relevant studies. The literature search and retrieval of publications were conducted following the PRISMA guidelines, as shown in Fig. 1 [25].

### 2.2 Inclusion and exclusion criteria

Based on Fig. 1, eligible papers were downloaded after the initial screenings. The following criteria apply to include articles published in this review study. Articles that did not match these standards were removed.

• Full-text available
• Cross-sectional studies
• Survey on AFM1 occurrences in cheese
• Articles are published in English or Persian language

### 2.3 Definitions and data extraction

Data for every research includes the first author's name, publication time, location of the research and sampling, type of cheese, sample size, detection method, the mean and standard deviation (SD) of AFM1 level, percentage of AFM1 in the contaminated sample, and the percentage of samples with AFM1 greater than the EC guideline for cheese was summarized and extracted. Because there is no access to the raw data, at the next step, data from each study was simulated using the mean, SD, and sample size of every research in Excel software (version 2016) by Equations (1), (2), (3)).

(1) $$Scaled\;mean=\ln\left({mean}^{2}/sqrt\left({mean}^{2}+{SD}^{2}\right)\right)$$

(2) $$Scaled\;SD=sqrt\left({\ln((mean}^{2}+{SD}^{2}\right)/{mean}^{2}))$$

(3) EXP=(NORMINV(RAND(),mean,SD)

Where; mean and SD is average AFM1 amount.

Eventually, the data obtained through the extraction process (comprising 2143 samples) based on the variables of cheese type, sample size, location of sampling, etc., were recorded in the SPSS software (version 21). Subsequently, descriptive parameters, including mean, SD, range, etc., were presented to provide overall results, a health risk assessment, and a comparison to the EC regulation.

### 2.4 Dietary exposure assessment

Equation (4) was employed to evaluate the average daily intake (ADI) of AFM1 via cheese ingestion [26], using daily cheese intake (DCHI) for Iranian children and adults, 8 and 13 kg per person per year, respectively [27]. The average concentration of AFM1 in cheese (C_AFM1_) and average body weight (BW) of children and adults, 20, 45.2, 78.7, and 74.5 kg for males aged <7, 7–16, 16–65, and >65 years old, and for the group of female, this value was considered to be equal to 15, 43.9, 65.5 and 66.3 kg, respectively [[28], [29], [30]].

(4) $$ADI\;\left(ng/kg\;bw/day\right)=\frac{C_{AFM1}\left(ng/kg\right)\times DCHI\;\left(kg\right)}{BW\;\left(kg\right)}$$

### 2.5 Health risk characterization

#### 2.5.1 Population risk for primary liver cancer

According to the Joint FAO/WHO Expert Committee on Food Additives (JECFA) report, cancer potency (CP) for exposure to 1 ngAFB1/kg bw/day in 100,000 population, resulting in upper boundaries for hepatitis B surface antigen-negative (HBsAg-) and hepatitis B surface antigen-positive (HBsAg+) populations are 0.049 and 0.562 additional cancer cases per 100,000, respectively [31]. On the other hand, it has been stated that AFM1's carcinogenic potency is one-tenth of AFB1, even in sensitive species such as Fisher rats and rainbow trout. The carcinogenic potency of AFM1 for HBsAg- and HBsAg + populations is 0.0049 and 0.0562 additional cancer cases per 100,000, respectively [31,32]. The prevalence of HBsAg+ in Iran is 1.5% [32,33]; therefore, to calculate CP in Equation (5), the population percentage (Pop) for HBsAg+ and HBsAg- is 0.015 and 0.985, respectively. Finally, the CP is 5.7 E −03 was considered.

(5) $$CP=\left(PHBs{Ag}^{+}\times \%PopHBs{Ag}^{+}\right)+\left(PHBs{Ag}^{-}\times \%PopHBs{Ag}^{-}\right)$$

Liver cancer risk (LCR) caused by exposure to AFM1 as a result of cheese consumption is calculated based on Equation (6) by multiplying the ADI by CP obtained in Equation (5) (5.7E-03), and it is reported as additional cases per year per 10^5^ populations [32]. Based on the criteria provided by the United States Environmental Protection Agency (USEPA), carcinogenic risk >10^−4^ is considered a severe carcinogenic risk and otherwise is acceptable (tolerable) [34].

(6) LCR=CP×ADI

#### 2.5.2 Margin of exposure (MOE)

According to equation (7), the benchmark dose (BMD) was divided by the ADI to assess the health risk of cheese consumption using the MOE. Based on a two-year study, BMD is the level of AFM1 determined to cause hepatocellular carcinoma in male Fischer rats and equals 570 ng/kg bw/day. A MOE value of 10,000 or higher would not be a serious public health concern [32,35,36].

(7) $$MOE=\frac{BMD\;\left(ng/kg\;bw/day\right)}{ADI\;\left(ng/kg\;bw/day\right)}$$

#### 2.5.3 Estimated hazard index (HI)

For further assessment of the safety of cheese consumption in Iran, the ADI was divided by an RFD (reference dose), and the HI was computed (equation (8)). The RFD is calculated by dividing the threshold dose (TD_50_) of AFM1 (10.38 μg/kg bw/day) by an uncertainty factor equal to 50000 (which creates a risk level of 1:100,000). TD_50_ is the level of AFM1 that causes cancers in 50% of experimental animals. Finally, the RFD value was considered equal to 0.2 ng/kg bw/day. The suggested value of HI = 1 and HI > 1 indicates that consumers are at significant health risk [26,32,37,38].

(8) $$HI=\frac{ADI\;\left(ng/kg\;bw/day\right)}{RFD\;\left(ng/kg\;bw/day\right)}$$

#### 2.5.4 Uncertainty and sensitivity analysis approach

The Monte Carlo simulation method was employed using 10,000 iterations on Oracle Crystal Ball software (version 11.1.2.4) within the Excel environment to conduct uncertainty analysis [29]. By employing this method and evaluating various values for the independent factors, namely C_AFM1_, BW, and DCHI, we completely evaluated all potential states of each of these variables, and the ADI calculation was iterated 100,000 times. “Then, by comparing the uncertainty upper bound (the 95th percentile) and the uncertainty lower bound (the 5th percentile) with the permissible limit of the decision-making parameters (10^−4^ for LCR, 10000 for MOE, and 1 for HI), the final decision was taken as to whether the cheese consumption was safe or hazardous”. The estimated ADI, LCR, MOE, and HI values were reported for the 5 and 95 percentiles in the current study. The calculations were performed to determine the probability distribution's median and 90% prediction intervals. The range of values falls between the 95th and 5th percentiles, providing the upper and lower boundaries, respectively. This review performed an uncertainty analysis on the mean AFM1 levels in cheese, irrespective of its kind, and it was based on the BW of children (32.6 ± 4.9 kg) [39] and adults (77.45 ± 13.6 kg) [29] linked to Iran.

### 2.6 Statistical analyses

This study used a one-way ANOVA test at the significance level (α = 0.05) to compare the average AFM1 in cheese by various kinds of cheese and sampling locations.

## 3 Results and discussion

In Table 1, the outcomes of previously published works on the discussed topic are summarized and presented. The occurrences of AFM1 in various varieties of cheese were studied in 22 studies. According to the studies conducted in most cities of Iran, the results can indicate the general contamination of different types of cheese consumed in Iran with AFM1.

**Table 1:** The results summary of previously published studies.

Reference	Study area	Sampling Locations	Samples analyzed, n	Cheese type	Analysis method	Contaminated samples, %	Exceed EC regulation (250 ng/kg), %	Amount (ng/kg)	
								Mean	S.D
[40]	Urmia	Market	40	Creamy	ELISA	100	NR	21.96	3.23
			40	White pasteurized	ELISA	100	NR	43.31	18.51
[2]	Esfahan and Yazd	Market	116	White pasteurized	ELISA	80.1	28.4	198.6	183.1
			94	Creamy		72.3	19.1	166.4	180.3
[7]	Esfahan	Market	100	White pasteurized	ELISA	52	8	169.1	32.4
[41]	Gilan	Market	90	White pasteurized	ELISA	86.66	23.33	151.97	NR
[16]	Esfahan	Retail stores	88	Traditional	ELISA	53.4	31.8	412	296
[42]	Ahvaz	Retail stores	61	White pasteurized	ELISA	60.7	60.7	103.6	54.1
[43]	Iranshahr	Supermarket	10	White pasteurized	ELISA	60	0	15.86	27.6

Reference	Study area	Sampling Location	Samples analyzed, n	Cheese type	Analysis method	Contaminated samples, %	Exceed EC regulation (250 ng/kg), %	Amount (ng/kg)	
								Mean	SD
[44]	Tehran	Supermarket	17	White pasteurized	ELISA	98.43	21	71.97	40.36
			21	White pasteurized				177.65	43.52
			10	Probiotic			0	29.52	22.27
			16	Probiotic				131.51	43.85
[45]	Tehran	Dairy factories	100	White pasteurized	ELISA	62	9.3	53.39	51.9
[46]	Tehran	Dairy factories	50	White pasteurized	ELISA	60	6	53.39	51.9
[47]	South Khorasan	Super market	43	Traditional	ELISA	32.55	27.9	29.9	4.77
			59	White pasteurized	ELISA	35	25	48.1	8.92
[20]	Shiraz	Dairy factories	22	White pasteurized	ELISA	100	9.09	82.61	NR
[27]	Hamadan	Market	40	White pasteurized	ELISA	97.5	10	115.16	79.22
			30	Creamy		93.3	10	141.2	77.06

Reference	Study area	Sampling Location	Samples analyzed, n	Cheese type	Analysis method	Contaminated samples, %	Exceed EC regulation (250 ng/kg), %	Amount (ng/kg)	
								Mean	S.D
[48]	Rafsanjan	Supermarket	45	White pasteurized	ELISA	64.4	20	135	28.2
			37	Lighvan		27	2.7	90.8	18.2
[37]	Kermanshah, Ilam, Hamadan, and Kurdistan	Rural regions	40	Traditional	ELISA and HPLC-FLD	65.5	10	158.4	86.0
[49]	Tehran, Esfahan, Shiraz and Yazd	Supermarkets and retail stores	72	White pasteurized	TLC	81.9	30.5	297	322.4
[50]	Southern Khorasan	Supermarket	19	Traditional	ELISA	63.2	31.6	4.77	NR
			15	Traditional		73.3	20	4.54	NR
			9	Traditional		66.7	33	5.6	NR
			19	White pasteurized		57.9	31.6	9.34	NR
			20	White pasteurized		70	20	9.04	NR
			20	White pasteurized		65	25	8.8	NR

Reference	Study area	Sampling Location	Samples analyzed, n	Cheese type	Analysis method	Contaminated samples, %	Exceed EC regulation (250 ng/kg), %	Amount (ng/kg)	
								Mean	SD
[26]	East and West Azarbaijan, Mazandaran, Gilan, and Fars	Rural regions	360	Traditional	ELISA	53.8	10.5	139	45.5
[51]	Hamedan	Market	188	White pasteurized	ELISA	70.7	36.2	343	328
[9]	Tehran, Esfahan, Tabriz and Shiraz	Dairy ranches	75	Lighvan	TLC	65.3	9.3	85	86.6
[52]	Mashhad	Dairy factories	129	White pasteurized	ELISA and HPLC	66.4	31.17	241.61	77.99
[14]	Mahabad	Livestock farm	12	Traditional	ELISA	100	33.33	198.24	91.55
			12	Traditional		100	16.6	134.75	69.2
			12	Traditional		100	8.33	175.07	86.33
			12	Traditional		100	8.33	137.3	61.7

**SD:** Standard deviation; **NR:** Not reported; **EU:** European Commission**; ELISA:** Enzyme-linked immunosorbent assay.

**SD**: Standard deviation; **NR**: Not reported; **EU**: European Commission; **ELISA**: Enzyme-linked immunosorbent assay.

S**SD:** Standard deviation; **NR:** Not reported; **EU:** European Commission**; ELISA:** Enzyme-linked immunosorbent assay.

**HPLC**: High-performance liquid chromatography; **FLD**: Fluorescence detection; **TLC**: thin layer chromatography.

**SD:** Standard deviation; **NR:** Not reported; **EU:** European Commission**; ELISA:** Enzyme-linked immunosorbent assay; **HPLC**: High-performance liquid chromatography; **TLC**: thin layer chromatography.

### 3.1 The analysis methods

The AFM1 analysis of dairy products using cost-effective, fast, and practical techniques has attracted researchers and regulators worldwide attention in recent years. Immunochemical methods like enzyme-linked immunosorbent assay (ELISA) and chromatography like high-performance liquid chromatography (HPLC) and thin layer chromatography (TLC) are frequently employed in the identification and quantification of AFM1 in milk and dairy products because of their reliability, selectivity, cost-effectiveness and high sensitivity to the lowest detection limits [28]. The findings indicate that between the various techniques of determining the amount of AFM1 in dairy products, ELISA, TLC, ELISA, and HPLC simultaneously and ELISA and HPLC-fluorescence detection (FLD) simultaneously, respectively in 18 (81.82%), 2 (9.09%), 1 (4.55%) and 1 (4.55%) studies have been used by researchers in various cities of Iran (Table 1). Because of the simplicity and no need for advanced features in the ELISA method, most research in this field in Iran is based on this detection method. It can be stated that the coordination of detection methods used for various types of cheese in Iran made it possible to compare their results in this study further.

Due to the variety of analytical techniques used, such as ELISA, HPLC, and TLD, for detecting AFM1 in dairy products, the results of different studies may report different concentrations [28]. In the works of Shahbazi et al. [26] and Bahrami et al. [53], higher concentrations of AFM1 were reported in the HPLC-FLD method than in ELISA, indicating a higher sensitivity of HPLC-FLD in the detection of AFM1, so it is suggested that HPLC-FLD be applied as a confirmation technique in the analysis of milk and dairy products in terms of AFM1in future studies.

### 3.2 The occurrence and concentration of AFM1 in cheese

Among the different dairy products, cheese is recognized as a rich source of distinct bioactive micro and macronutrients that can play a significant role in human health [26]. However, cheese may be more critical than other dairy products for intaking AFM1 because of the AFM1's preferential affinity to the casein portion of cheese, the increase in the contact surface during production, and the concentration of AFM1 in cheese being 3 to 5 times higher than in the milk itself [37,54].

It has been shown that *Aspergillus* fungus produces AFM1 in cheese, possibly due to the presence of AFM1 in the base milk or in the milk powder used to make the cheese [2]. AFM1 level in the cheese may vary according to the cheese type, the production conditions, and the level of milk contamination by the AFM1 [26]. Therefore, the results of this study show that the mean concentration of AFM1 between different types of cheese in Iran is significantly different (P < 0.001) (Table 2). The overall mean concentration of AFM1 for cheese in Iran was 160 ± 175 ng/kg. So for white pasteurized, traditional, creamy, probiotic, and Lighvan, the average AFM1 was 171 ± 190, 164 ± 162, 126 ± 150, 92.04 ± 58, and 86 ± 75, respectively (Table 2). Considering that traditional cheese production in Iran is increasing due to consumer awareness that traditional foods are free of artificial additives [26], the relatively high mean of AFM1 in this type of cheese in this study can concern health authorities. Continuous and more basic monitoring of this dairy product is suggested [55]. Moreover, nomads and villagers often produce traditional cheeses under relatively poor sanitary conditions, which can increase the formation of toxic compounds [37].

**Table 2:** The final finding of AFM1 in consumed cheese in Iran based on sampling location and cheese type.

Sampling location/Cheese type	Samples analyzed, n	Contaminated samples, %	Exceed regulation, n (%)^a^	Mean ± SD^b^ (ng/kg)	Range (ng/kg)	P
Sampling location						
Dairy factories	301	72.1%	52 (17%)	136 ± 116	2–607	<0.001
Markets	1319	69.25%	223 (16%)	175 ± 211	ND c-2236	
Livestock farms	523	83.51%	25 (4%)	135 ± 63	8.06–496.07	
Cheese type						
White pasteurized	1219	72.03%	191 (15%)	171 ± 190	ND-2236	<0.001
Traditional	622	73.49%	82 (13%)	164 ± 162	ND-1845	
Creamy	164	88.53%	23 (14%)	126 ± 150	9–1214	
Probiotic	26	98.43%	0 (0%)	92.04 ± 58	7–167	
Lighvan	112	46.15%	4 (3%)	86 ± 75	8.06–496.07	
Total	2143	72.42%	300 (13%)	160 ± 175	ND-2236	

a The EC and ISIRI limit for AFM1 is 250 ng/kg for cheese.

b Standard deviation.

c Not detection.

The findings illustrated in Table 2 demonstrate that out of 2143 samples of various types of cheese in different studies, 301 (14.04%) samples were from dairy factories, 1319 (61.54%) samples from dairy stores and markets and 523 (24.4%) samples were collected and analyzed from livestock farms and rural areas. The current research outcomes also show that the average concentration of AFM1 among samples gathered from various places has a significant difference (P < 0.001). So, the average AFM1 for cheese samples collected from dairy factories, markets, and livestock farms was 136 ± 116 ng/kg, 175 ± 211 ng/kg, and 135 ± 63 ng/kg, respectively (Table 2).

The variation in AFB1 levels in the fodder of dairy species is due to reasons such as climatic conditions, ecological, qualitative, and economic characteristics of the farm, different conditions of feed storage in terms of air circulation, hotness, moisture, and storage time, and various forms of livestock forage, can be the primary factor contributing to the variation in the concentration of AFM1 in different types of cheese and sample collection locations [28,56,57]. Ghaneian et al. [57] reported that severe fungal contamination, including hyphomycetes black and saprophytes, was detected due to poor feed storage conditions in Yazd, Iran. The studies of Makhdoumi et al. [58], Bahrami et al. [37], and Shahbazi et al. [26] in Iran, and the study of Ismaiel et al. [59] in Egypt reported higher concentrations of AFM1 in cheese samples collected in winter compared to summer. The best way to reduce the contamination of milk and dairy products with AFM1 is to control the feed of lactating species in terms of contamination with AFB1. Aflatoxin-producing fungi grow on fodder and grains in hot and humid climatic conditions, including a minimum ambient temperature of 25 °C, adequate oxygen, and humidity of more than 15% [56,57,60,61].

In the study of Iqbal & Asi [62] in Pakistan, the mean AFM1 concentration of white and creamy cheeses was 147.8 ± 11.3 ng/kg and 102.6 ± 13.4 ng/kg, respectively. In the Temamogullari & Kanici [63] study in Turkey, the mean AFM1 concentration was 103.2 ± 29.13 ng/kg for white pickled cheese. In the Malissiova et al. [64] study in Greece, the mean AFM1 concentration of feta cheese was 2.98 ± 0.72 ng/kg. In the Dashti et al. [65] study in Kuwait, the mean AFM1 concentration of white cheese was 87.6 ng/kg, and in the study of Daou et al. [66] in Lebanon, the mean AFM1 concentration of cheeses was 85 ng/kg. From the point of view that in all these studies, the mean level of AFM1 for various varieties of cheese is lower than in the studies from Iran, it can be concluded that the level of AFM1 in cheese in Iran is greater than in several neighboring and non-neighboring countries.

Based on the obtained results, overall, 72.42% of 2143 different cheese samples were contaminated with AFM1, so for white pasteurized, traditional, creamy, probiotic, and Lighvan, this amount was 72.03%, 73.49%, 88.53%, 98.43%, and 46.15%, respectively (Table 2). Based on the different locations of cheese samples collected in different studies for dairy factories, markets, and livestock farms, this contamination was obtained as 72.1%, 69.25%, and 83.51%, respectively (Table 2). In the study of Amer & Ibrahim [67], 33% of various kinds of cheese were found to be contaminated with AFM1 in Egypt; another study conducted by Öztürk Yilmaz & Altinci [68] found that AFM1 was present in 40% of white cheese in Turkey; similarly, Bakırdere et al. [69] found that AFM1 was present in 38% of cream cheese and 53% of white cheese in Turkey, in another study, Dashti et al. [65] found that AFM1 was present in 80% of white cheese in Kuwait, and Malissiova et al. [64] found that AFM1 was present in 28% of the feta cheese that was produced in Greece.

Among the samples of cheese contaminated with AFM1, 300 (13%) samples had AFM1 concentrations greater than EC standard (Table 2). Moreover, Makhdoumi et al. [58] reviewed that 17.8% of the cheeses consumed in Iran have an AFM1 concentration higher than the standard set by the EC. The difference can be due to the broader range of studies in the present study. Based on sampling locations, dairy factories, markets, and livestock farms, 52 (17%), 223 (16%), and 25 (4%) samples had AFM1 concentrations higher than the EC regulation. On the other hand, this amount is based on the type of cheese examined for white pasteurized, traditional, creamy, probiotic, and Lighvan was 191 (15%), 82 (13%), 23 (14%), 0 (0%) and 4 (3%) respectively (Table 2). Regarding the prevalence of samples exceeding EC regulatory limits, certain recent research conducted in different countries confirms the findings of the current study, while other studies do not demonstrate such concurrence. In the study of Iqbal & Asi [62] in Pakistan, 15% and 11% of white cheese and cream cheese samples, respectively, in Dashti et al. [65] in Kuwait, 26.4% of white cheese, in Amer & Ibrahim [67] in Egypt, 33% different cheeses and the study of Ardic et al. [70] in Turkey, 26.4% of white brined cheese samples had AFM1 concentration higher than the EC regulation.

### 3.3 Exposure and risk characterization

#### 3.3.1 AFM1 intake by cheese consumption

The amount of average daily intake (ADI) of AFM1 calculated for different age groups of males and females is presented in Table 3. Based on the obtained results, in general, the ADI of AFM1 through the consumption of different cheese types for males in the age group <7, 7–16, 16–65, and >65 years was 0.18, 0.13, 0.07, and 0.08 ng/kg bw/day, respectively, for female in the same age group, it was 0.23, 0.13, 0.09 and 0.09 ng/kg bw/day, respectively. Considering the lower average amount of AFM1 in Lighvan cheese compared to other cheeses, the results show that this cheese has the lowest ADI for consumers compared to other cheeses, and white pasteurized cheese has the highest amount. Based on the sampling location, the results show that the cheese purchased from the market has the highest amount of ADI of AFM1 for consumers, and if it is from livestock farms and rural areas, it has the lowest amount (Table 3).

**Table 3:** The ADI of AFM1 via Cheese consumption in Iran based on various age groups for males and females.

Sampling location/Cheese type		Age groups for males (Year)				Age groups for females (Year)			
		<7	7–16	16–65	>65	<7	7–16	16–65	>65
		ADI (ng/kg bw/day)							
Sampling location	Dairy factories	0.15	0.11	0.06	0.07	0.20	0.11	0.07	0.07
	Markets	0.19	0.14	0.08	0.08	0.26	0.14	0.10	0.10
	Livestock farms	0.15	0.11	0.06	0.07	0.20	0.11	0.07	0.07
Cheese type	White pasteurized	0.19	0.14	0.08	0.08	0.25	0.14	0.09	0.09
	Traditional	0.18	0.13	0.08	0.08	0.24	0.13	0.09	0.09
	Creamy	0.14	0.10	0.06	0.06	0.18	0.10	0.07	0.07
	Probiotic	0.10	0.07	0.04	0.04	0.13	0.08	0.05	0.05
	Lighvan	0.09	0.07	0.04	0.04	0.13	0.07	0.05	0.05
Total		0.18	0.13	0.07	0.08	0.23	0.13	0.09	0.09

**Fig. 2:**
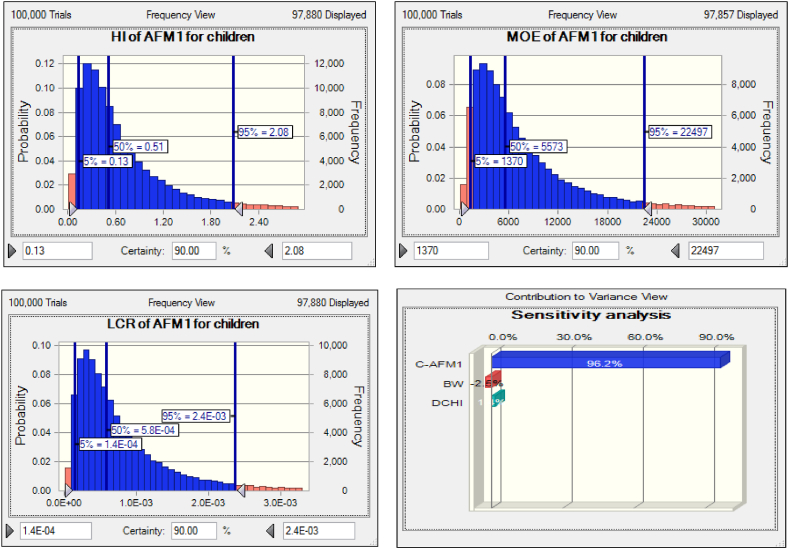
Uncertainty and sensitivity analysis of input variables related to AFM1via cheese consumption for Iranian children.

**Fig. 3:**
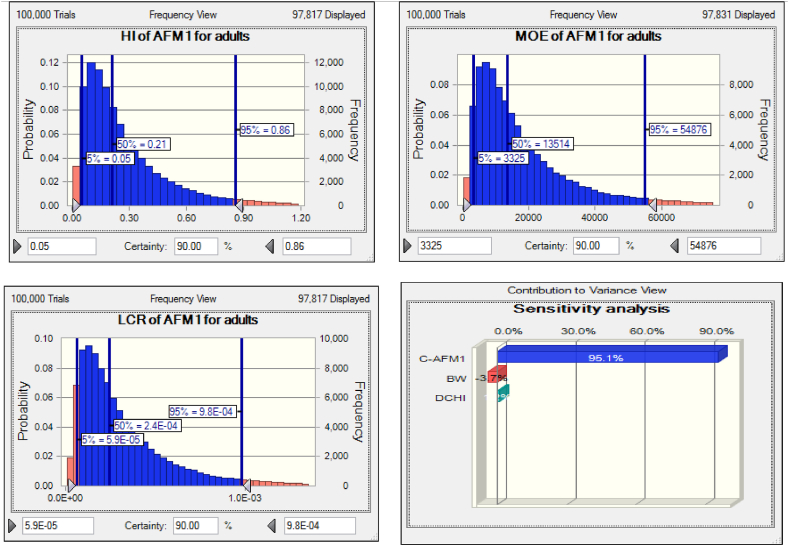
Uncertainty and sensitivity analysis of input variables related to AFM1via cheese consumption for Iranian adult.

The difference in parameters influencing the ADI, including the concentration of C_AFM1_, BW, and DCHI, can cause a difference in the ADI of AFM1. The results of this study's sensitivity analysis show that the three abovementioned independent parameters had the greatest impact on the ADI of the AFM1 due to cheese ingestion in Iran for adults and children is C-AFM1 > BW > DCHI, respectively (Fig. 2, Fig. 3). Alteration of C-AFM1 levels through unfavorable AFB1 growth conditions appears to be the most practical way of controlling or reducing AFM1 in dairy products [38].

In the study of Malissiova et al. [64] in Greece, the ADI of AFM1 for Feta cheese was reported to be 0.01 ng/kg bw/day, which is lower than the lowest ADI obtained (0.04) in the current study. According to a review study by Marimón Sibaja et al. [71], the ADI of AFM1 as a result of milk consumption in Brazil, Costa Rica, Colombia, and Mexico based on studies published from 2003 to 2018 was 2.4, 1.0, 1.2, and 20.9 ng/kg bw/day, respectively. According to the research of Bahrami et al. [37], ADI of AFM1 was reported as 0.018 and 0.249 when inesting raw milk and dairy products in summer and winter, respectively. Moreover, Shahbazi et al. [26] found that the ADI of AFM1 through cheese consumption was 0.03 and 0.04 b y the ELISA method in the summer and winter seasons and 0.04 and 0.05 b y the HPLC-FLD method, respectively. The summer and winter seasons have created different environmental conditions for producing AFB1 and AFM1, which has affected the ADI of AFM1.

#### 3.3.2 Human health risk assessment

The dietary risk assessment estimates the risk caused by consuming food containing xenobiotics and the relationship between possible food chain risks and human health risks [32]. The health risk of exposure to AFM1 for Iranian cheese consumers in various age groups was determined using the LCR, MOE, and HI approaches.

The results obtained for LCR show that the LCR related to AFM1 for males in the age groups of <7, 7–16, 16–65, and >65 years are 1.0E-03, 7.3E-04, 4.2E-04, and 4.4E-04 and for female in the same age group it is 1.3E-03, 7.5E-04, 5.0E-04 and 5.0E-04 additional cancer cases/year/10^5^ population (Table 4). Hence, it is imperative to implement suitable strategies to mitigate the levels of AFM1 in different types of cheese and sanitary control of the cheese production process in factories and rural areas. Hasninia et al. [29] also revealed that the amount of LCR for milk consumers was greater than 0.0001 in most investigated cases, similar to the present study. Also, Hooshfar et al. [32] noted that the concentration of LCR linked to AFM1 in formula-fed infants aged below 6 months was measured to be 0.00010. Pardakhti & Maleki [72] informed the levels of LCR resulting from milk ingestion in various cities in Iran, including “Tehran, Mashhad, Ahwaz, Babol, Isfahan, Kermanshah, Miandoab, Hamedan, and Urmia” were found to be 5.7E-05, 6.3E-05, 1.2E-04, 2.8E-04, 6.9E-04, 3.3E-03, 6.9E-06, 3.6E-06 and 2.1E-02, respectively, (in terms of additional cancer cases/year/105 population). Based on the LCR results for cheese in Iran in this study, the carcinogenic potential of AFM1 for cheese consumers in Iran can be described as a health hazard.

**Table 4:** The LCR and MOE of AFM1 via cheese consumption in Iran based on various age groups for male and female.

Sampling location/Cheese type		Age groups for males (Year)				Age groups for females (Year)			
		<7	7–16	16–65	>65	<7	7–16	16–65	>65
		LCR (Additional cancer cases/Year/10^5^ population)							
Sampling location	Location 1	8.5E-04	6.2E-04	3.5E-04	3.7E-04	1.1E-03	6.4E-04	4.3E-04	4.2E-04
	Location 2	1.1E-03	7.9E-04	4.6E-04	4.8E-04	1.5E-03	8.2E-04	5.5E-04	5.4E-04
	Location 3	8.5E-04	6.1E-04	3.5E-04	3.7E-04	1.1E-03	6.3E-04	4.2E-04	4.2E-04
Cheese type	White pasteurized	1.1E-03	7.8E-04	4.5E-04	4.7E-04	1.4E-03	8.0E-04	5.4E-04	5.3E-04
	Traditional	1.0E-03	7.4E-04	4.3E-04	4.5E-04	1.4E-03	7.7E-04	5.1E-04	5.1E-04
	Creamy	7.9E-04	5.7E-04	3.3E-04	3.5E-04	1.1E-03	5.9E-04	3.9E-04	3.9E-04
	Probiotic	5.8E-04	4.2E-04	2.4E-04	2.5E-04	7.7E-04	4.3E-04	2.9E-04	2.8E-04
	Lighvan	5.4E-04	3.9E-04	2.2E-04	2.4E-04	7.2E-04	4.0E-04	2.7E-04	2.7E-04
Total		1.0E-03	7.3E-04	4.2E-04	4.4E-04	1.3E-03	7.5E-04	5.0E-04	5.0E-04
Sampling location/Cheese type		MOE							
Sampling location	Location 1	3810	5262	9162	8673	2858	5111	7626	7719
	Location 2	2961	4090	7120	6740	2221	3972	5926	5999
	Location 3	3838	5301	9230	8738	2879	5149	7682	7776
Cheese type	White pasteurized	3030	4185	7287	6898	2273	4065	6065	6139
	Traditional	3160	4364	7598	7193	2370	4238	6324	6401
	Creamy	4113	5680	9890	9362	3084	5517	8231	8331
	Probiotic	5630	7776	13538	12816	4222	7552	11268	11405
	Lighvan	6025	8322	14489	13716	4519	8082	12059	12206
Total		3239	4473	7788	7372	2429	4344	6482	6561

The results presented in Table 4 also show that the MOE values for males and females in all age groups are generally less than 10,000. This amount for males in the age group <7, 7–16, 16–65, and >65 years was 3239, 4473, 7788, and 7372, respectively, and for females in the same age group was 2429, 4344, 6482 and 6561 respectively, which demonstrates that the consumption of cheese in Iran in all age groups is of public concern and should be taken seriously as a health priority for risk management measures [35]. Hooshfar et al. [32] demonstrated that the MOE for babies below 6 months of age who ingest formula was 7671.6, matching the findings of the current analysis. Moreover, Hasninia et al. [29] also reported that the P95 of the MOE of AFM1 in summer <10,000 for males and females ingesting raw milk in all age groups. Considering that ADI influences MOE, it is suggested that deeper monitoring and control be done in producing and processing milk and cheese in terms of AFB1 and AFM1 in farms and dairy factories in Iran.

Based on the results, in general, the amount of HI through the consumption of various kinds of cheese for males in the age group <7, 7–16, 16–65, and >65 years was 0.9, 0.6, 0.4, and 4.4 respectively, and for female in the same age group, it was 1.2, 0.7, 0.4 and 0.4 respectively (Table 5). The overall results show that there is a worrying health risk for females in the children age group (<7 years) as a result of exposure to AFM1 in cheese in Iran (HI > 1). Moreover, based on the cheese type, in females in the age group of children, consumption of white pasteurized and traditional cheese can increase the health risk; also, based on the location of purchase of cheese, the consumption of cheese purchased from markets can increase the health risk for female, in the age group of children (Table 5).

**Table 5:** The HI of AFM1 via Cheese consumption in Iran based on various age groups for males and females.

Sampling location/Cheese type		Age groups for males (Year)				Age groups for females (Year)			
		<7	7–16	16–65	>65	<7	7–16	16–65	>65
		HI.							
Sampling location	Dairy factories	0.7	0.5	0.3	0.3	1.0	0.6	0.4	0.4
	Markets	1.0	0.7	0.4	0.4	1.3	0.7	0.5	0.5
	Livestock farms	0.7	0.5	0.3	0.3	1.0	0.6	0.4	0.4
Cheese type	White pasteurized	0.9	0.7	0.4	0.4	1.3	0.7	0.5	0.5
	Traditional	0.9	0.7	0.4	0.4	1.2	0.7	0.5	0.4
	Creamy	0.7	0.5	0.3	0.3	0.9	0.5	0.3	0.3
	Probiotic	0.5	0.4	0.2	0.2	0.7	0.4	0.3	0.2
	Lighvan	0.5	0.3	0.2	0.2	0.6	0.4	0.2	0.2
Total		0.9	0.6	0.4	0.4	1.2	0.7	0.4	0.4

The sensitivity analysis results indicate that the 95th percentile value for HI of AFM1 via cheese intake is alarming for children (2.08) but not for adults (0.86) (Fig. 2, Fig. 3). It is necessary for the health of children who consume cheese in Iran to control the parameters influencing HI, including ADI and AFM1, followed by AFB1, to take more effective measures like the ones mentioned above. Malissiova et al. [64] reported HI = 0.05 for Feta cheese collected from the market in Greece, which was much lower than the lowest level (0.2) reported in the current work.

Bahrami et al. [37] indicated that HI through raw milk and dairy product intake in the summer and winter seasons was reported to be 0.54 and 1.254, respectively; also, Hasninia et al. [29] revealed a higher rate of HI in winter compared to summer for male and female ingesting raw milk in the age groups of 7–16, 16–65 and > 65 which show the impact of weather conditions on increasing HI levels.

The overall findings of risk assessment in terms of HI demonstrate that the health risk caused by exposure to AFM1 in cheese in Iran for females in the age group of children is in an inappropriate range and predicts a worrying health risk for this group. Moreover, based on the MOE and LCR parameters, the situation is very alarming for all age groups of males and females. It highlights the need to pay particular attention to the production and preparation of cheese in Iran to ensure consumer safety.

## 4 Conclusions and recommendations

This study investigated the concentration, prevalence, and health risk assessment (HRA) for AFM1 in cheese consumed in Iran. The overall mean AFM1 for cheese in Iran was 175 ± 160 ng/kg, lower than the EC regulation limit (250 ng/kg). The highest amount of AFM1 based on the type of cheese was related to white pasteurized (171 ± 190 ng/kg) and based on the sampling location related to markets (175 ± 211 ng/kg). In light of the findings, there is a notable variation in the average concentration of AFM1, dependent upon the variety of cheese and the sampling location. The observed variations can be attributed to AFB1 production resulting from the various processing conditions employed in producing fodder and cheese. The health conditions of milk concerning AFB1 are contingent upon many regional parameters. Based on the results, it was found that among the contaminated samples with AFM1, generally, 13% of the samples had AFM1 higher than EC regulation. In addition, in terms of the health risk arising from the consumption of cheese in Iran, based on the LCR, MOE, and HI parameters, it was found that the situation is relatively alarming; this issue holds particular importance, particularly in the setting of infants. Accordingly, preventing the development of environments favorable to the growing of various *Aspergillus* species that produce AFB1 in livestock fodder is the most significant fundamental step that can be taken to lower the levels of AFM1 in Iranian cheese.

To reduce the toxic levels of AFM1 and subsequent adverse health effects caused by cheese consumption in Iran, industrial and traditional dairy producers should be educated about livestock fodder rearing and storage principles. Training in sanitary production and processing of dairy products according to world standards is suggested for industrial and traditional cheese producers across Iran. The study's findings suggest several measures to reduce AFM1's toxic levels and prevent adverse health effects caused by cheese consumption in Iran. These measures include educating both industrial and traditional dairy producers on proper animal feed rearing and storage principles, regular inspection of dairy plants by experts, adhering to standard principles of cheese processing and packaging, equipping factory laboratories for routine monitoring and detection of toxins, developing precise detection methods, and raising awareness among health authorities about the risks posed by AFM1 in disrupting food safety standards are suggestions for the health of cheese consumers in Iran.

## Author contribution statement

All authors listed have significantly contributed to the development and the writing of this article.

## Data availability statement

No data was used for the research described in the article.

## Declaration of competing interest

The authors declare that they have no known competing financial interests or personal relationships that could have appeared to influence the work reported in this paper.
